# GenomeFLTR: filtering reads made easy

**DOI:** 10.1093/nar/gkad410

**Published:** 2023-05-13

**Authors:** Edo Dotan, Michael Alburquerque, Elya Wygoda, Dorothée Huchon, Tal Pupko

**Affiliations:** The Shmunis School of Biomedicine and Cancer Research, George S. Wise Faculty of Life Sciences, Tel Aviv University, Tel Aviv 69978, Israel; The Shmunis School of Biomedicine and Cancer Research, George S. Wise Faculty of Life Sciences, Tel Aviv University, Tel Aviv 69978, Israel; The Shmunis School of Biomedicine and Cancer Research, George S. Wise Faculty of Life Sciences, Tel Aviv University, Tel Aviv 69978, Israel; School of Zoology, George S. Wise Faculty of Life Sciences, Tel Aviv University, Tel Aviv 69978, Israel; The Steinhardt Museum of Natural History, Israel National Center for Biodiversity Studies, Tel-Aviv University, Tel Aviv 69978, Israel; The Shmunis School of Biomedicine and Cancer Research, George S. Wise Faculty of Life Sciences, Tel Aviv University, Tel Aviv 69978, Israel

## Abstract

In the last decade, advances in sequencing technology have led to an exponential increase in genomic data. These new data have dramatically changed our understanding of the evolution and function of genes and genomes. Despite improvements in sequencing technologies, identifying contaminated reads remains a complex task for many research groups. Here, we introduce GenomeFLTR, a new web server to filter contaminated reads. Reads are compared against existing sequence databases from various representative organisms to detect potential contaminants. The main features implemented in GenomeFLTR are: (i) automated updating of the relevant databases; (ii) fast comparison of each read against the database; (iii) the ability to create user-specified databases; (iv) a user-friendly interactive dashboard to investigate the origin and frequency of the contaminations; (v) the generation of a contamination-free file. Availability: https://genomefltr.tau.ac.il/.

## Graphical Abstract

**Graphical Abstract:**
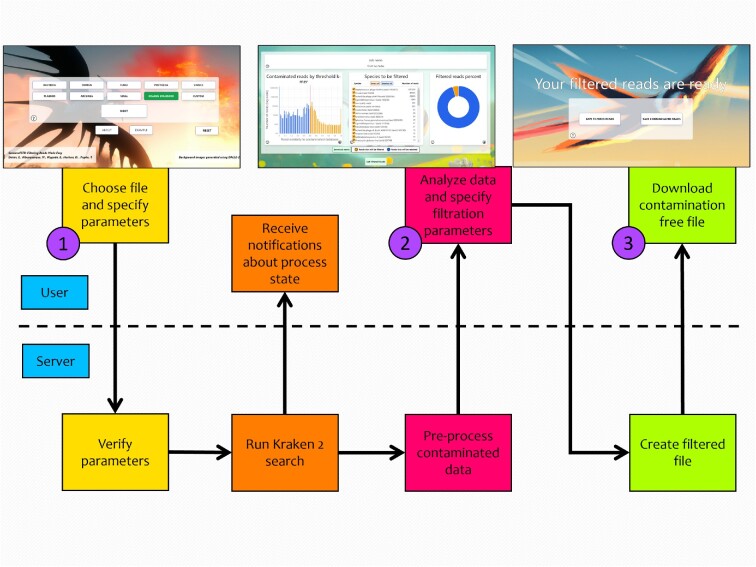

## INTRODUCTION

Sequencing costs are constantly decreasing (1). Research groups are now able to generate large sequence datasets from various organisms, and hence the size of GenBank doubles every few months (2). These data drive discoveries in ecology, evolution, molecular biology, and medicine (3–5). Detecting and filtering contaminant DNA is a main challenge when processing next-generation sequencing (NGS) data. Contaminant reads are defined as reads that originated from an organism different from the one that the researchers aimed at sequencing. Read contamination can have a significant effect on downstream analyses, such as false positive single-nucleotide polymorphisms (SNP) identification (6), incorrect labels on sequences in metagenomic studies (7) and inaccurate phylogenetic inference (8).

Previous studies have shown that some of the most used biological datasets contain a large proportion of contaminated sequences. For example, over two billion contaminated sequences were detected in RefSeq and over 14,000 putative contaminants were identified in the non-redundant (NR) database (9). Moreover, repeated elements characterizing human cells were found in a quarter of non-primate genomes available in NCBI (the National Center for Biotechnology Information) (10). As these datasets are often taken as ‘ground truth’, filtering contaminated sequences is of high importance prior to most bioinformatic analyses (11,12).

Previous contaminant-detection algorithms can be classified by various criteria (13). One main criterion is the presence or absence of reference databases to detect contaminations. Methodologies that do not rely on reference databases, search for read-specific features such as low-complexity and low-quality scores (14). When searching for bacterial contaminants, the following features were searched for: atypical GC content, presence of intron-less genes, and small scaffolds (15,16). Regarding algorithms that rely on reference databases for detecting contaminations, several search for the presence of a few single-copy gene markers [e.g. (17,18)]. These methods are aimed to detect the presence of additional copies of these markers indicating the presence of contaminations and possibly identify their sources. However, these gene marker-based methods aim at detecting contaminations and not at filtering a dataset from contaminants. Other algorithms, using reference databases take a genome-wide approach to detect contaminations. With these algorithms, filtration may take place pre- or post-assembly (13). One advantage of post-assembly methods is that they can take synteny into account to detect contaminant sequences (12). However, it is likely that the assembly itself can be improved by removing contaminated reads prior to the assembly. Contamination-detection algorithms are often tuned to specific taxa, e.g. the tool GUNC searches for lack of phylogenetic homogeneity across prokaryotic contigs (19). Several pre-assembly methods rely on splitting the reads into small fragments and finding similarities against specific datasets using BLAST (20). Great progress was achieved by the development of efficient algorithms for mapping short DNA segments to genomes, which allows classifying reads to taxonomic units (21–24). Such fast approaches are a prerequisite for the development of efficient web servers for the detection and filtering of contaminated reads and contigs.

The above algorithms, as well as additional tools developed by specific research groups (21,25), require downloading the pipeline components (e.g. scripts, programs), downloading and maintaining databases, and may require heavy computer clusters (i.e. multi-CPUs) and technological skills. Here we present GenomeFLTR, a web server that easily filters genomic reads. No technical skill, downloading, or computational power is needed. Raw reads are uploaded to the server and contaminated reads are removed, based on similarity to databases that are periodically and automatically updated. A user can also provide a tailored dataset to compare against. The contaminated reads are analyzed, e.g. the reads taxonomy distribution is provided. Our server provides a simple and interactive graphical user interface (GUI) that allows controlling the filtering process (Video 1, Supplementary data).

## MATERIALS AND METHODS

### Input

The sole mandatory input for the GenomeFLTR web server is a file (or two files for paired reads, see below), containing the reads to be filtered. Standard formats such as Fastq and Fasta are accepted. In addition, a user has to select a database against which the reads are queried (e.g. to detect bacterial contaminants, a user can choose a bacterial database containing multiple genomes from a diverse set of bacteria). A user may also input a custom database (see below). Finally, a user may specify an email to which the results link will be sent.

### Database

The entire set of sequence databases available in GenomeFLTR is automatically updated monthly from NCBI. These databases are processed for the Kraken search engine format (21). We also allow users to choose the database against which to compare their read data. Default databases are bacteria, human, fungi, protozoa, univec (i.e. a dataset of vector sequences), plasmid, archaea, viral, Kraken standard (i.e. all complete bacterial, archeal, and viral genomes in Refseq), and custom. For the custom database, a user inserts the NCBI taxonomy identifiers of the species included in RefSeq (NCBI Reference Sequence Database) to compare against and may choose specific accession numbers of genomes from this species to analyze. If accession numbers are not provided, the first three genomes from RefSeq are downloaded for each species. To download the genomes for the custom database we use a script available at https://github.com/kblin/ncbi-genome-download.

### Search engine

Each read is first split into *k*-mers (*k*-mers are substrings of the read with length *k*; for example, 3-mer for the read: ‘ATGG’ will be: ‘ATG’ and ‘TGG’). To maximize both speed and accuracy, we use the Kraken 2 search engine (21) to query each *k*-mer (with *k* = 35) against the selected database. A phylogenetic tree representing the evolutionary relationships within the taxon included in each Kraken database is used to classify hits to either species or ancestral nodes. If a *k*-mer only matches a single species, it will be assigned to it. If a *k*-mer matches multiple species, it will be assigned to the most recent common ancestral node of all these species. Note that different *k*-mers within the same read might be assigned to different nodes of the phylogenetic tree. The output of this step is a file containing, for each of the reads, a list of species or ancestral nodes and the number of *k*-mers matched to each node.

### Read classification

**Figure 1.**
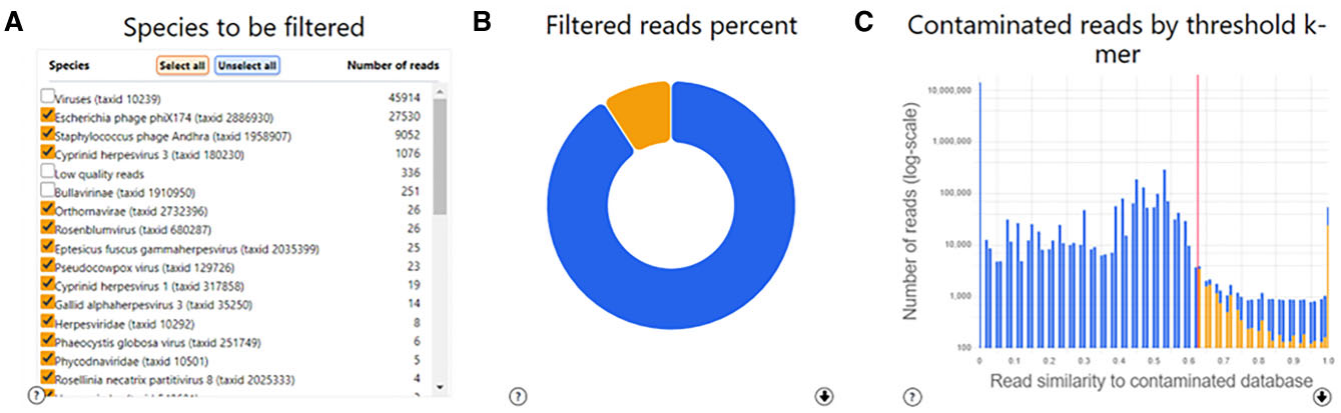
GenomeFLTR dashboard. The dashboard contains the following interactive panels: (**A**) a tabular description of the nodes and their corresponding number of reads. Species can be included or excluded from the list of contaminated species by marking a checkbox; (**B**) a pie chart representing the percent of current contaminated and retained reads; (**C**) a histogram showing the number of reads for each of the read-contamination scores. Low values correspond to reads with low similarity to the selected database. The red line represents the currently selected threshold to filter by. Pressing on the graph will update the threshold. The dashboard is interactive, i.e. the different panels are updated according to the user's actions.

The output of the previous step is further processed in order to classify each read to a specific node in the tree. To this end, for each read and for each node we define a read-node score, which is the number of *k*-mers mapped to this node divided by the total number of *k*-mers possible for that read (*l* – *k* + 1, where *l* is the read length). For each read, we identify the node that maximizes the read-node score and assign the read to this node. A tabular description (Figure 1A) of the number of contaminated reads from each node is provided as interactive visual output by GenomeFLTR as well as a pie chart indicating the percentage of contaminated reads (Figure 1B).

### Determining which reads to filter

We also define a read-contamination score, which is the sum, over all nodes of the tree, of the read-node score. This score quantifies the percent of *k*-mers that were mapped to the contaminated database out of the *l* – *k* + 1 total *k*-mers. The higher the read-contamination score, the more likely it is that the read is a contamination and hence should be filtered. A histogram illustrating the distribution of the read-contamination score is given as an interactive graphical output by GenomeFLTR (Figure 1C). The user specifies a threshold cutoff that determines which reads will be labeled as contamination and which will be retained in the ‘clean’ data. By default, this threshold is set to 0.5. This threshold can be set interactively by clicking on the bars of the histogram. Reads with a score lower than the threshold (this threshold is marked by a red line in the graphical plot) are colored blue and will be retained, while reads colored orange will be filtered once the user presses the ‘Get filtered results’ button. Of note, reads that do not match any of the genomes in the database are also part of the clean data.

It is possible that a user chooses to retain reads of specific species. For example, if a user sequenced a metagenomic sample containing multiple bacteria species, and would like to retain only a subset of those bacteria, e.g. bacteria that are known to exist in a specific niche. He can do so, by choosing specific species to retain / filter from the interactive tabular section of the GUI. The pie chart and the histogram are updated accordingly in real-time. We note that in this case, some blue reads (retained reads) could appear to the right of the red bar, which indicates the read-contamination score threshold.

### Obtaining contamination-free data

Pressing the ‘Get filtered results’ button initiates the post process, which iterates over the reads and identifies the ‘cleaned’ from the contaminated ones. When the post process is finished, a link to download a compressed file (i.e. a ‘.gz’ file) containing all the non-contaminated reads is provided on the screen and via email to the user.

### Implementation

GenomeFLTR is implemented in Python using the Flask framework. The source code is available at: https://github.com/michaelalb/GenomeFltr. The web server submits jobs that are processed on ProLiant XL170r Gen9, equipped with 128 GB RAM and 28 CPU cores per node. Background images were generated using Dall-E 2 (26).

### Paired-end files

Another feature implemented in the web server is the filtering of paired-end reads. Each end is first processed independently as described above. Next, the node-score of the pair-end read is the maximum over the two ends. For example, if one end has a read-node score of 0.2 for species X, and the other end the read-node score is 0.75 to species Y, the result of the paired read is a read-node score of 0.75 to species Y. Based on the read scores, the paired reads are either filtered or not, thus, if one end within a pair is considered to be a contamination, the entire paired-end read is discarded.

### Data structure

We transform the list of the reads (which contain millions of reads) into a matrix, in which rows are bins of the read-node score (101 bins: 0, 0.01, …, 1) and columns are nodes in the tree. Each cell denotes the number of reads for that bin and node. This data structure allows us to present interactive graphs in real-time.

### Multiple filtration steps

It may be beneficial to filter reads first against the univec database and then to filter the obtained clean data against, e.g. bacterial contaminants. Iterative executions allow breaking down the filtration procedure, thus cleaning the data against combinations of pre-existing categories. Such an approach is demonstrated in the case study below.

## CASE STUDY

We present the GenomeFLTR output by analyzing the transcriptome reads available under accession number SRR1300899. This paired-read dataset originated from a myxozoan parasite (*Kudoa iwatai*). Myxozoans are microscopic eukaryotic parasites of fish, with a large negative economic impact (27). Because of their small size and their presence within fish tissues, we expected to find fish reads as well as some bacterial reads and possibly a small number of human reads in these NGS data. This parasitic dataset was published before the fish host genome was available and thus these data were not filtered before their submission to public repositories (28). We analyzed a total of 50 million paired-end reads (100 million reads in total) from these data in two steps. First, we excluded the fish reads by performing a custom filtering analysis in which we provided the taxonomic id (taxid 8175) of the host fish as input. The program automatically downloaded the corresponding Refseq genome GCF_900880675.1 for this analysis. GenomeFLTR inferred that ∼17.3% of the reads (8,641,393 paired reads) were of fish origin using a read similarity threshold above 0.75. We then downloaded the remaining uncontaminated reads and conducted a second filtering analyses against the Kraken standard database (again with a threshold of 0.75). The remaining read data contained bacterial contaminations from various sources, for example Proteobacteria (39,129 paired reads) and *Staphylococcus* (49,243 paired reads). It also contained a number of reads from human origin (107,658 paired reads). Using the web server option to mark nodes that should not be filtered, we decided not to filter cellular organisms and root (taxid 1), which reflect reads that are potentially of eukaryotic origin and thus may be genuine myxozoan reads. In total, 1.22% of the reads were filtered, generating contamination-free data that are ready for further analyses.

## DATA AVAILABILITY

GenomeFLTR is freely available without registration or login requirements at https://genomefltr.tau.ac.il/.

## Supplementary Material

gkad410_Supplemental_Files
