# Supplementary material for: GenomeFLTR: filtering reads made easy
Source: Nucleic Acids Res. 2023 May 13;51(W1):W232–6. doi: 10.1093/nar/gkad410 (PMC10320065; doi:10.1093/nar/gkad410)

SUPPLEMENTARY DATA

GenomeFLTR: Filtering Reads Made Easy

Edo Dotan[1,†], Michael Alburquerque[1,†], Elya Wygoda[1,†], Dorothée Huchon[2,3,*], Tal Pupko[1,*]

[1] The Shmunis School of Biomedicine and Cancer Research, George S. Wise Faculty of Life Sciences, Tel Aviv University, Tel Aviv 69978, Israel.

[2] School of Zoology, George S. Wise Faculty of Life Sciences, Tel Aviv University, Tel Aviv 69978, Israel.

[3] The Steinhardt Museum of Natural History, Israel National Center for Biodiversity Studies, Tel-Aviv University, Tel Aviv 69978, Israel

[†] Joint Authors

* To whom correspondence should be addressed:

Tal Pupko, Tel: +972 3 640 7693; E-mail: talp@tauex.tau.ac.il

* Correspondence may also be addressed to:

Dorothée Huchon, Tel: +972 3 640 9817; E-mail: huchond@tauex.tau.ac.il

VIDEO

Video 1. GenomeFLTR process. First, the user uploads the file containing the reads and specifies the required parameters. Next, the user controls the interactive GUI to filter the contaminated reads. Finally, the contamination-free file is ready to download.

Still image to represent video in the print PDF:

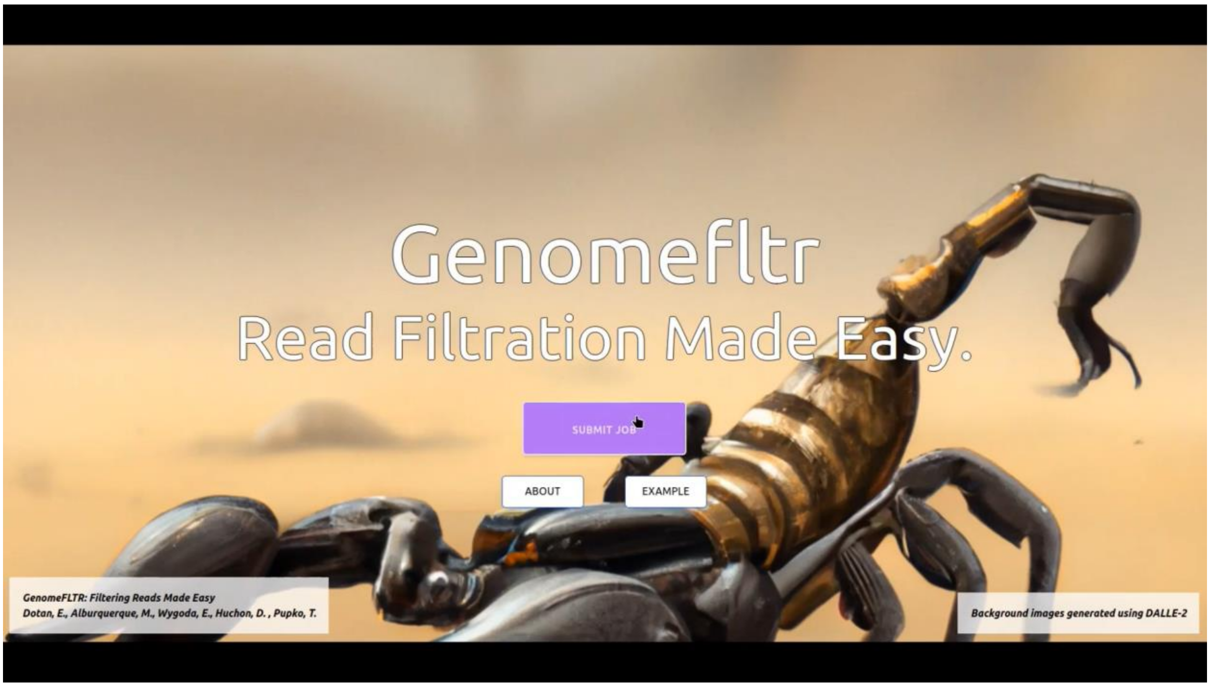

Supplement: gkad410_Supplemental_Files [file gkad410_supplemental_files.zip › GenomeFLTR_supp.pdf]
